# Added value of zoomed-echo-planar imaging diffusion-weighted imaging for evaluation of periampullary carcinomas

**DOI:** 10.1007/s00261-023-03990-2

**Published:** 2023-07-05

**Authors:** Jingjing Liu, Mengyue Huang, Yanan Ren, Man Xu, Jinxia Zhu, Yinhua Li, Jingliang Cheng

**Affiliations:** 1 Department of MR Imaging, The First Affiliated Hospital of Zhengzhou University, Zhengzhou, China; 2 MR Collaboration, Siemens Healthcare Ltd., Beijing, China

**Keywords:** Diffusion-weighted imaging, Zoomed, Periampullary carcinomas

## Abstract

**Background:**

To assess the image quality feasibility and diagnostic value of zoomed diffusion-weighted imaging (z-EPI DWI) using echo-planar imaging (EPI) compared with conventional DWI (c-EPI DWI) in patients with periampullary disease.

**Methods:**

Thirty-six patients with periampullary carcinomas and fifteen with benign periampullary disease were included in this study. All the subjects underwent MR cholangiopancreatography (MRCP), c-EPI DWI, and z-EPI DWI. Two radiologists independently assessed image quality of the two image sets, including overall image quality and lesion conspicuity. In addition, signal intensity and ADC measurements of DWIs in the periampullary lesions were conducted. Diagnostic accuracies of the combined image sets of MRCP and z-EPI DWI were compared with those of a combined set of MRCP and c-EPI DWI.

**Results:**

z-EPI DWI showed significantly better image quality scores (anatomic structure visualization, 2.94 ± 0.24; overall image quality, 2.96 ± 0.17) compared to that with c-EPI DWI (anatomic structure visualization, 2.02 ± 0.22; overall image quality, 2.04 ± 0.24) (both *P* < 0.01). For all the periampullary malignant lesions and small lesions (≤ 20 mm), there was better delineation of lesion conspicuity and the lesion margin, as well as diagnostic confidence with z-EPI DWI (all *P* < 0.05). The rate of periampullary malignancy’s hyperintense signal on z-EPI DWI was increased to 91.7% (33/36) compared to c-EPI DWI (69.4% (25/36)) (*P* = 0.023). For all malignant lesions and small lesions, the diagnostic accuracy scores were increased using the MRCP and z-EPI DWI combined set, compared to the MRCP and c-EPI DWI combined set (*P* < 0.05). Diagnostic accuracy for detection and differentiation of malignant lesions from benign lesions significantly improved for the MRCP and z-EPI DWI combined set compared with MRCP and c-EPI DWI combined set (*P* < 0.05). There were no significant differences between c-EPI DWI and z-EPI DWI in the ADC values of periampullary malignant and benign lesions (*P* > 0.05).

**Conclusions:**

z-EPI DWI has an advantage that could lead to remarkable image quality improvements and enhanced lesion visualization of periampullary carcinomas. z-EPI DWI was superior to c-EPI DWI for detecting, delineating, and diagnosing the lesions, particularly for small challenging lesions.

**Graphical abstract:**

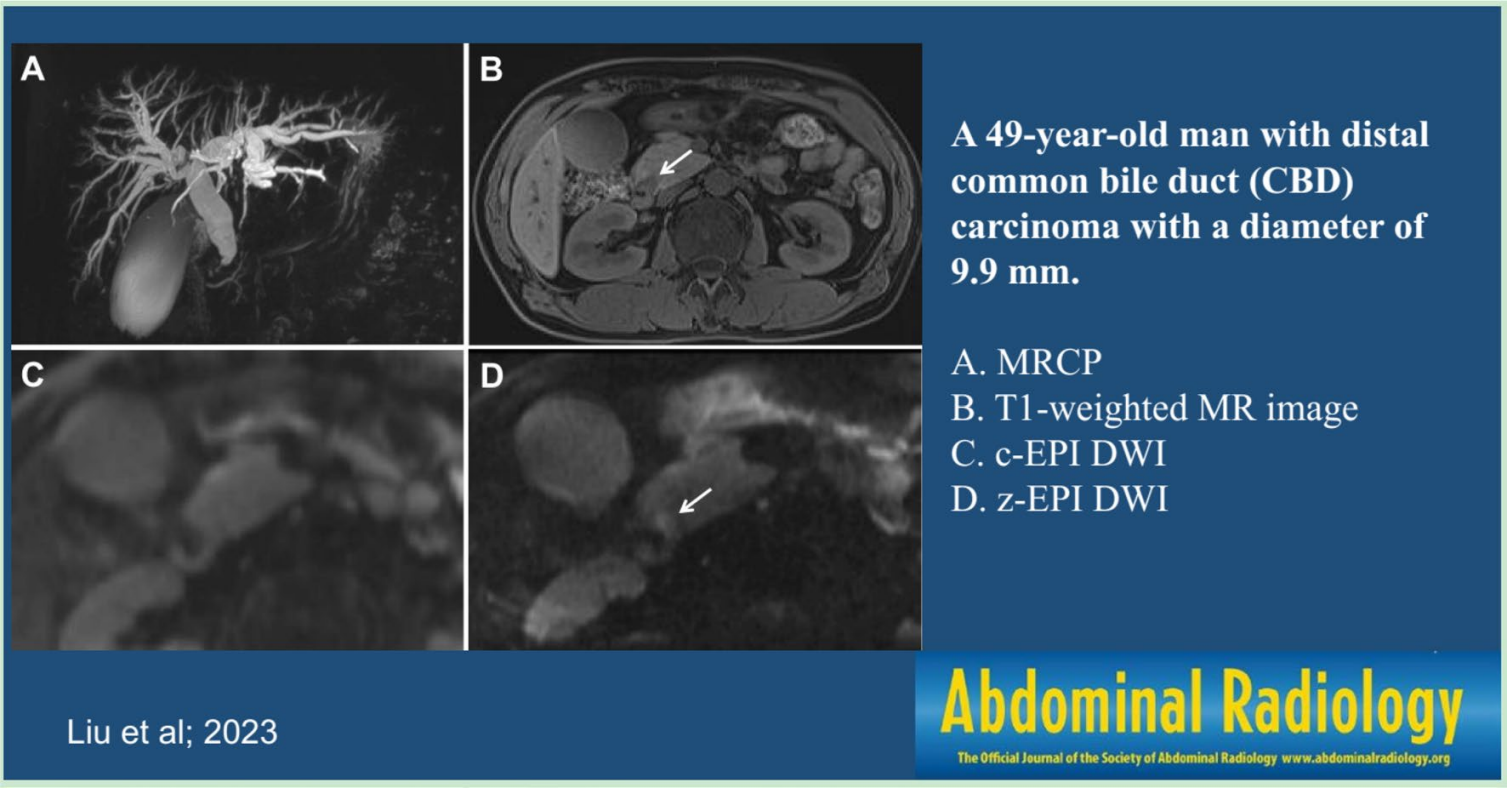

## Introduction

It is a common finding during magnetic resonance imaging (MRI) that the bile ducts down to the level of the periampullary region could be dilated due to various benign or malignant lesions. While it is often difficult to make a definitely diagnosis based on MRI, an endoscopy and histopathological confirmation is still required in most cases. Differentiation of benign from malignant periampullary lesions is not always satisfactory based on conventional MR imaging alone because of the small tumor size and an overlap in imaging features. In clinical practice, low diagnostic confidence in the diagnosis of a benign periampullary lesion means that a malignant periampullary lesions cannot be excluded, which might lead to a large number of incidence rate and and mortality related to unnecessary surgery. Accurate diagnosis and exclusion of benign lesion with high diagnostic confidences are crucial.

Diffusion-weighted imaging (DWI), which can provide functional information concerning the status of tissue cellularity [1–5], has been used with abdominal MRI to detect and characterize various abdominal lesions, particularly malignancies. The application of DWI in distinguishing benign and malignant bile duct strictures has been investigated in several studies [2, 6, 7]. However, implementation of DWI using single-shot echo-planar imaging (EPI) in patients with biliary disease is challenging due to artifacts, particularly those arising from differences in tissue susceptibility, poor spatial resolution, and inherent image blurring [8–10].Therefore, when using conventional DWI, accurately diagnosing benign and malignant lesions in the periampullary area, detecting and clearly diagnosing small periampullary malignant tumors, especially those without dilation of the bile duct, may be a challenge. According to recent studies, compared with conventional EPI (c-EPI) DWI, two-dimensional spatially selective radiofrequency excitation pulses combined with reduced field of view (FOV) (“zoomed”) along phase-encoding directions could lead to superior image quality with reduced spatial distortions and artifacts and improved identification of small anatomic structures [11–14]. This new technology allows to focus on the organ of interest instead of unnecessary imaging of the entire upper abdominal space. Several studies have demonstrated the value of z-EPI DWI in evaluating pancreas, kidney, prostate, uterine, spinal cord, rectum, and head and neck tumors [11–20]. The application of zoomed EPI (z-EPI) DWI as a complement to c-EPI DWI may help detect and diagnose periampullary diseases, but research has not yet been conducted.

## Methods

### Study population

**Fig. 1:**
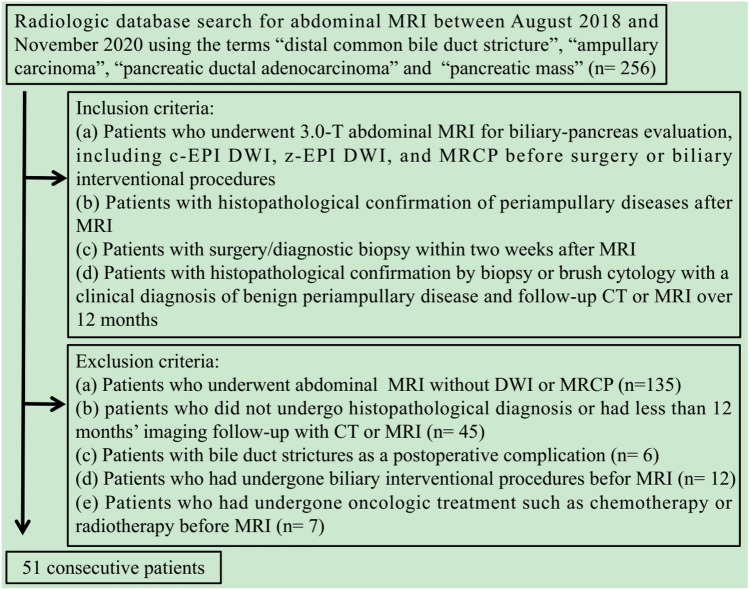
Flow chart of the study population

This retrospective study was approved by the institutional ethics review board with a waiver of patients’ informed consent. We searched our radiologic database for abdominal MR examinations performed between August 2018 and November 2020 by using the search terms “distal common bile duct stricture,” “ampullary carcinoma,” “pancreatic ductal adenocarcinoma,” and “pancreatic mass,” and the search yielded 256 patients. We used the following inclusion criteria: (a) patients who underwent 3.0-T abdominal MRI for biliary-pancreas evaluation, including c-EPI DWI, z-EPI DWI, and MRCP before surgery or biliary interventional procedures, (b) patients with histopathological confirmation of periampullary diseases after MRI, (c) patients with surgery/diagnostic biopsy within two weeks after MRI, (d) patients with histopathological confirmation by biopsy or brush cytology with a clinical diagnosis of benign periampullary disease and follow-up CT or MRI over 12 months. On the basis of these inclusion criteria, 205 patients were excluded (Fig 1). Finally, 51 patients were included in this study. Fifteen patients (mean age, 61.4 ± 15.8 years, range 18–79; 9 men and 6 women) had benign periampullary disease and 36 patients (mean age, 60.8 ± 10.5 years, range 39–80; 18 men and 18 women) had periampullary malignancy.

Thirty-six lesions (tumor size 21.69 ± 10.05 mm (4.00–45.7 mm)) in 36 patients with periampullary malignancy were evaluated in our study population, and 20 small lesions with a diameter less than or equal to 20 mm (tumor size 14.78 ± 4.38 mm (4.00–20.00 mm)) were included. Of these, 25 patients underwent surgery, and 11 patients underwent only diagnostic biopsy; the findings included 13 histologically confirmed pancreatic adenocarcinomas, 15 distal common bile duct cholangiocarcinomas, four ampullary carcinoma, and four duodenal adenocarcinomas.

Of these patients with benign periampullary disease, one patient underwent surgery, and five patients underwent only diagnostic biopsy, revealing two histologically confirmed ampulla of Vater tubular adenomas, three distal common bile duct inflammatory stenosis cases, and one case with inflammation of the descending segment of the duodenum. Eight patients underwent ERCP, confirming seven common bile duct stones or sludge and one descending duodenal diverticulum. One patient was clinically diagnosed with autoimmune pancreatitis. Clinical and demographic data for the two groups are summarized in Table 1.

**Table 1:** Clinical and demographic data

Parameter	Periampullary malignancy (*n* = 36)	Periampullary benignancy (*n* = 15)
Age (Year)		
Average	60.8 ± 10.5	61.4 ± 15.8
Range	39–80	18–79
Gender		
Male	18	9
Female	18	6
The size of lesions (mm)		
Average	21.69 ± 10.05	
Range	4.00–45.7	
Diameter ≤ 20 mm (mm)		
Average	14.78 ± 4.38	
Range	4.00–20.00	
Confirm		
Confirmed by pathology		
Surgery	25	1
Biopsy	11	5
Confirmed by clinic	0	9
Diagnosis		
Pancreatic adenocarcinomas	13	
Distal common bile duct cholangiocarcinomas	15	
Ampullary cancers	4	
Duodenal adenocarcinomas	4	
Ampulla of Vater tubular adenomas		2
Distal common bile duct inflammatory stenosis		3
Inflammation of the descending segment of the duodenum		1
Common bile duct stones		7
Descending duodenal diverticulum		1
Autoimmune pancreatitis		1
Quantitative ADC Measurements	29	4

### MRI technique

An upper abdomen MRI study of 51 patients was performed on a 3T whole-body MR system (MAGNETOM Prisma, Siemens Healthcare, Germany) with an 18-channel phased-array body coil as the receiver coil. Both a c-EPI DWI (b values = 50 and 800 sec/mm^2^) and z-EPI DWI (b values = 50 and 800 sec/mm^2^) of the periampullary region in the same patient were obtained. For the z-EPI DWI, a two-dimensional spatially selective RF pulse using an echo-planar transmit trajectory was applied. The z-EPI imaging parameters of DWI were as follows: 2000/61 (repetition time (ms)/echo time (ms)), 5-mm slice thickness, 230 mm × 120-mm field of view, 1.5 × 1.5 × 5 reconstructed voxel size (mm^3^), and 154 × 50 matrix, acquisition time was 3min20s~5min45s, depending on breathing pattern (respiration control: trigger). The c-EPI imaging parameters of DWI were as follows: 4500/56 (repetition time (ms)/echo time (ms)), 5-mm slice thickness, 350 mm × 292 mm field of view, 2.2 × 2.2 × 5 reconstructed voxel size (mm^3^), 158 × 121 matrix, and 1 min 52 s acquisition time (respiration control: Free-breathing). Both a breath-hold single-section 2D MRCP and navigator-triggered 3D MRCP were obtained, and the parameters were as follows: 2D MRCP: repetition time (ms)/echo time (ms), 4500/735; flip angle, 180°; slice thickness, 50mm; matrix,384×268; field of view, 300mm×300 mm; bandwidth,352-Hz/pixel; echo space(msec) 6.5ms. 3D MRCP: repetition time (ms)/echo time (ms), 2400/702; flip angle, 140°; slice thickness, 1.2mm; matrix,384×384×276; field of view, 350 mm×350 mm; bandwidth, 350-Hz/pixel; echo space(msec) 5.1 ms. The conventional sequences included a coronal breath-hold T2-weighted half-Fourier single-shot turbo spin echo sequence (HASTE) (1400/67 (repetition time (ms)/echo time (ms)), 5-mm slice thickness, 360mm × 360-mm field of view, 256 × 256 matrix), an axial fat-suppressed respiratory triggered (RT) T2-weighted turbo spin echo sequence (TSE) (3100/87 (repetition time (ms)/echo time (ms)), 5-mm slice thickness, 380mm × 380-mm field of view, 320 × 320 matrix), and a three-dimensional volumetric interpolated breath-hold examination (VIBE) sequence (3.9/1.89 (repetition time (ms)/echo time (ms)), 3-mm slice thickness, 380mm × 309-mm field of view, 288 × 187 matrix) was repeated four times for the T1-weighted dynamic contrast-enhanced (DCE) imaging (pre-enhanced phase, arterial phase, portal vein phase, and delay phase). After pre-enhanced phases, 0.1 mmol/kg of Gd-DTPA was injected at a rate of 2 mL/s. Thirty-two patients underwent DCE MRI.

### Qualitative image analysis

All the qualitative image analyses were performed independently on a PACS workstation by two experienced radiologists (with 5 and 8 years of experience in abdominal MRI, respectively) in a randomized fashion. The reviewers were blinded to the patients’ information including the pathology and clinical diagnosis. Each reader ranked the z-EPI DWI and c-EPI DWI in terms of image quality, considering the anatomic structure visualization, artifacts, and overall image quality. The anatomic structure visualization, artifacts, and overall image quality of the DWI images acquired with both EPI techniques were evaluated according to a 4-point scale: (1) anatomic structure visualization (1, poorly visualized anatomy and non-diagnostic; 2, fairly delineated periampullary region with margin blurring; 3, good delineation of periampullary region with a sharp margin; and 4, excellent sharpness of periampullary region); (2) artifacts (1, severe and non-diagnostic; 2, moderate; 3, mild; and 4, absent); and (3) overall image quality (1, poor image quality, considered non-diagnostic; 2, fair image quality, somewhat impairing diagnostic quality; 3 good image quality, not impairing diagnostic quality; and 4, excellent image quality).

Both EPI techniques were evaluated by using a 4-point scale for lesion conspicuity (1, lesion not detectable; 2, merely recognizable lesion-to-background contrast; 3, intermediate lesion-to-background contrast or high contrast with indistinct lesion margin; and 4, excellent lesion-to background contrast and a clear lesion margin) and lesion margin (1, lesion margin not detectable; 2, obscure; 3, indistinct; and 4, distinct). Diagnostic confidence was evaluated according to a 4-point scale based on the combination of DWI and conventional T_1_WI (T1-weighted imaging) and T_2_WI (T2-weighted imaging) (1, DWI was not useful for confirming the diagnosis of malignant periampullary lesions as the lesion characterization on DWI was indeterminate or the lesion was invisible; 2, lesion characterization on DWI was consistent with the confirmed diagnostic impression on conventional imaging; 3, DWI helped to confirm the suspected diagnosis on conventional imaging; and 4, DWI helped characterize the lesion as malignant when the conventional imaging findings were indeterminate for characterization or the lesion was invisible).

Signal intensity assessment of the periampullary lesions on the two DWI image sets was also conducted. The signal intensity of the periampullary lesions visually assessed compared with the signal intensity of the liver on a 4-point scale using a b-value of 800 sec/mm^2^ was as follows: 0, isointense; 1, slightly hyperintense; 2, significantly hyperintense and 3 hypointense. Criteria for malignant periampullary lesions on DWI were defined as lesions showing hyperintensity. Criteria for malignant periampullary lesions on MRCP images were defined if the stricture was characterized by an eccentric and abrupt narrowing with irregular margins of the distal parts of the bile duct and/or association with the double-duct sign. Criteria for benign periampullary lesions on MRCP images were the smooth and gradual tapering of the distal parts of the bile duct. For the MRCP images, the probability of malignancy for the distal biliary stricture was rated using a 5-point scale: 1, definitely benign; 2, probably benign; 3, indeterminate; 4, probably malignant; and 5, definitely malignant. The sensitivity calculations were based on only those lesions awarded a confidence rating of 4 or 5.

Diagnostic accuracy was compared between the combined set of MRCP and c-EPI DWI and that of MRCP and z-EPI DWI. The two radiologists recorded the possibility of malignant periampullary lesions with a consensus using a 5-point confidence rating scale, as follows: 1, definitely benign (benign on MRCP without DWI hyperintensity); 2, probably benign (indeterminate on MRCP without DWI hyperintensity); 3, indeterminate (benign on MRCP with DWI hyperintensity and malignant on MRCP without DWI hyperintensity); 4, probably malignant (indeterminate on MRCP with DWI hyperintensity); and 5, definitely malignant (malignant on MRCP with DWI hyperintensity). The sensitivity calculations were also based on those lesions, awarding a confidence rating of 4 or 5. Readers first evaluated only c-EPI DWI images and, subsequently, the z-EPI DWI using the same criteria. For each EPI DWI, only high b-value images (b = 800 sec/mm^2^) were analyzed.

### Quantitative analysis

Quantitative measurements of the ADC values of malignant and benign periampullary lesions were independently performed by a single different radiologist with 6 years of experience in radiology. The radiologist was blinded to the patients’ information including the pathology and clinical diagnosis. The ADC values of the periampullary lesions were obtained by manually placing a circular region of interest (ROI) on the ADC maps acquired from both the c-EPI and z-EPI DWI sequences. ROIs were placed at near-identical locations on both sequences with care to avoid vessels, cysts, bile ducts, and pancreatic ducts. Effort was made to have 3 ROIs in the lesions. For some lesions, it was difficult to accurately measure the ADC values of most benign strictures and some malignant lesions because of their relatively small sizes. Therefore, quantitative analysis was performed in 29 patients with periampullary malignancy and 4 patients with benign periampullary lesions.

### Statistical analysis

We performed all statistical analyses using SPSS Statistics 19 (IBM, Armonk/NY, USA). A *P* value < 0.05 was considered to be statistically significant. The Wilcoxon signed-rank test was used between c-EPI DWI and z-EPI DWI for comparing the qualitative image analysis scores. For periampullary disease, lesion conspicuity, lesion margin, and diagnostic confidence were compared with the estimates obtained. In addition, diagnostic accuracy scores were compared between the MRCP and c-EPI DWI combined set and the MRCP and z-EPI DWI combined set. The Fisher’s exact test was used between c-EPI DWI and z-EPI DWI for comparing visual assessment of DWI in the periampullary lesions. The area under the ROC curve (AUC) was calculated to determine the diagnostic accuracy between the MRCP and c-EPI DWI combined set and the MRCP and z-EPI DWI combined set. Comparisons were made using the average scores between the two readers. Inter-reader agreement for each assessed qualitative evaluation was assessed using weighted κ statistics. Inter-reader agreement was considered as slight for κ = 0.00–0.20, fair for *κ* = 0.21–0.40, moderate for *κ* = 0.41–0.60, substantial for *κ* = 0.61–0.80, and almost perfect for *κ* = 0.81–1.00. ADC values of periampullary lesions were also compared between the two DWI sequences using the Wilcoxon signed-rank test.

## Results

### Image quality scores analysis

Compared to c-EPI DWI, z-EPI DWI showed significantly better image quality scores (Table 2). With z-EPI DWI, the periampullary region presented better anatomic structure visualization (2.94 ± 0.24) and overall image quality (2.96 ± 0.17), compared to that with c-EPI DWI (anatomic structure visualization, 2.02 ± 0.22; overall image quality, 2.04 ± 0.24) (both *P* < 0.01). Artifacts were improved on z-EPI DWI (2.92±0.27) (c-EPI DWI, 2.81±0.44) (*P* = 0.039).

**Table 2:** Comparison of image quality scores between c-EPI and z-EPI diffusion-weighted imaging sequences (b = 800 sec/mm^2^)

	Anatomic structure visualization	Artifacts	Overall image quality
z-EPI			
Reader1	2.94 ± 0.24	2.92 ± 0.27	2.94 ± 0.24
Reader2	2.94 ± 0.24	2.92 ± 0.27	2.98 ± 0.14
Average	2.94 ± 0.24	2.92 ± 0.27	2.96 ± 0.17
c-EPI			
Reader1	2.04 ± 0.28	2.80 ± 0.45	2.00 ± 0.20
Reader2	2.00 ± 0.20	2.82 ± 0.43	2.08 ± 0.34
Average	2.02 ± 0.22	2.81 ± 0.44	2.04 ± 0.24
*P* value*	*P* < 0.001	0.039	*P* < 0.001

Data are mean ± standard deviation

*z-EPI* zoomed echo-planar imaging; *c-EPI* conventional echo-planar imaging

*Wilcoxon signed-rank test was performed between c-EPI and z-EPI DWI sequences using averaged image quality scores of two readers

### Lesion scores analysis

For all the periampullary malignant lesions (*n* = 36), there was better delineation of lesion conspicuity and the lesion margin, as well as diagnostic confidence with z-EPI DWI (all *P* < 0.05) (Table 3). The malignant lesions with z-EPI DWI showed better detection and delineation with higher lesion conspicuity (2.80 ± 0.61), lesion margins (2.79 ± 0.59), and diagnostic confidence (2.60 ± 0.68), compared to those with c-EPI DWI (lesion conspicuity, 2.01 ± 0.73; lesion margin, 1.78 ± 0.50; diagnostic confidence, 2.14 ± 0.87) (all *P* < 0.05). Furthermore, periampullary malignant lesions with a diameter less than or equal to 20 mm (*n* = 20) were evaluated in terms of above three parameters between the two DWI techniques, and the malignant lesions with z-EPI DWI showed better detection and delineation with higher lesion conspicuity (2.95 ± 0.48), lesion margins (2.90 ± 0.48), and diagnostic confidence (2.73 ± 0.64), compared to those with c-EPI DWI (lesion conspicuity, 1.93 ± 0.82; lesion margins, 1.73 ± 0.60; and diagnostic confidence, 1.90 ± 0.91) (all *P* < 0.05). However, there was no statistically significant differences between all malignant lesions and small lesions for lesion scores of lesion conspicuity, lesion margins, and diagnostic confidence from the two image datasets (all *P* > 0.05). For benign periampullary lesions, there were no statistically significant differences between the two image datasets for lesion conspicuity, lesion margin, or diagnostic confidence (all *P* > 0.05), which may be related to the small number of included benign lesions, small size lesions, and most of benign periampullary lesions showed isointense signal on DWI.

**Table 3:** Comparison of lesion scores between c-EPI and z-EPI sequences (b = 800 sec/mm^2^)

	Periampullary malignancy (*n* = 36)						Periampullary Benignancy (*n* = 15)		
	All lesions (*n* = 36)			Small lesions (≤20 mm) (*n* = 20)					
	Lesion conspicuity	Lesion margin	Diagnostic confidence	Lesion conspicuity	Lesion margin	Diagnostic confidence	Lesion conspicuity	Lesion margin	Diagnostic confidence
z-EPI									
Reader1	2.83 ± 0.65	2.75 ± 0.60	2.58 ± 0.69	3.00 ± 0.56	2.85 ± 0.49	2.70 ± 0.66	1.33 ± 0.72	1.33 ± 0.72	1.40 ± 0.83
Reader2	2.78 ± 0.59	2.83 ± 0.61	2.61 ± 0.69	2.90 ± 0.45	2.95 ± 0.51	2.75 ± 0.64	1.33 ± 0.72	1.33 ± 0.72	1.40 ± 0.83
Average	2.80 ± 0.61	2.79 ± 0.59	2.60 ± 0.68	2.95 ± 0.48	2.90 ± 0.48	2.73 ± 0.64	1.33 ± 0.72	1.33 ± 0.72	1.40 ± 0.83
P value#				0.27	0.434	0.532			
c-EPI									
Reader1	2.03 ± 0.77	1.78 ± 0.48	2.14 ± 0.87	1.95 ± 0.89	1.70 ± 0.57	1.90 ± 0.91	1.20 ± 0.41	1.33 ± 0.72	1.33 ± 0.72
Reader2	2.00 ± 0.72	1.78 ± 0.54	2.14 ± 0.87	1.90 ± 0.79	1.75 ± 0.64	1.90 ± 0.91	1.20 ± 0.41	1.33 ± 0.72	1.33 ± 0.72
Average	2.01 ± 0.73	1.78 ± 0.50	2.14 ± 0.87	1.93 ± 0.82	1.73 ± 0.60	1.90 ± 0.91	1.20 ± 0.41	1.33 ± 0.72	1.33 ± 0.72
*P* value*	*P* < 0.001	*P* < 0.001	0.008	0.001	*P* < 0.001	0.008	0.157	0.157	0.317
*P* value#				0.694	0.742	0.337			

Data are mean ± standard deviation

*z-EPI* zoomed echo-planar imaging; *c-EPI* conventional echo-planar imaging

*Wilcoxon signed-rank test was performed between c-EPI and z-EPI DWI sequences using averaged lesion scores of two readers

^#^Wilcoxon signed-rank test was performed between all malignant lesions and small lesions using averaged lesion scores of two readers

### Inter-reader agreement analysis

Overall inter-observer agreement between the two readers was moderate to almost perfect, and weighted κ between the readers ranged from 0.485 to 1 for z-EPI DWI and from 0.477 to 1 for c-EPI DWI, respectively (Table 4).

**Table 4:** Inter-reader agreement of image quality scores and lesion scores

	z-EPI (b = 800 sec/mm^2^)	c-EPI (b = 800 sec/mm^2^)
Anatomic structure visualization	1.000 (1.000, 1.000)	0.793 (0.000, 1.000)
Artifacts	1.000 (1.000, 1.000)	0.931 (0.738, 1.000)
Overall image quality	0.485 (0.000, 1.000)	0.477 (0.000, 0.850)
Lesion conspicuity	0.924 (0.802, 1.000)	0.908 (0.804, 1.000)
Lesion margin	0.883 (0.751, 1.000)	0.925 (0.809, 1.000)
Diagnostic confidence	0.969 (0.900, 1.000)	1.000 (1.000, 1.000)

Data in parentheses are 95% confidence intervals

*z-EPI* zoomed echo-planar imaging; *c-EPI* conventional echo-planar imaging

### Signal of lesions on DWI

**Fig. 2:**
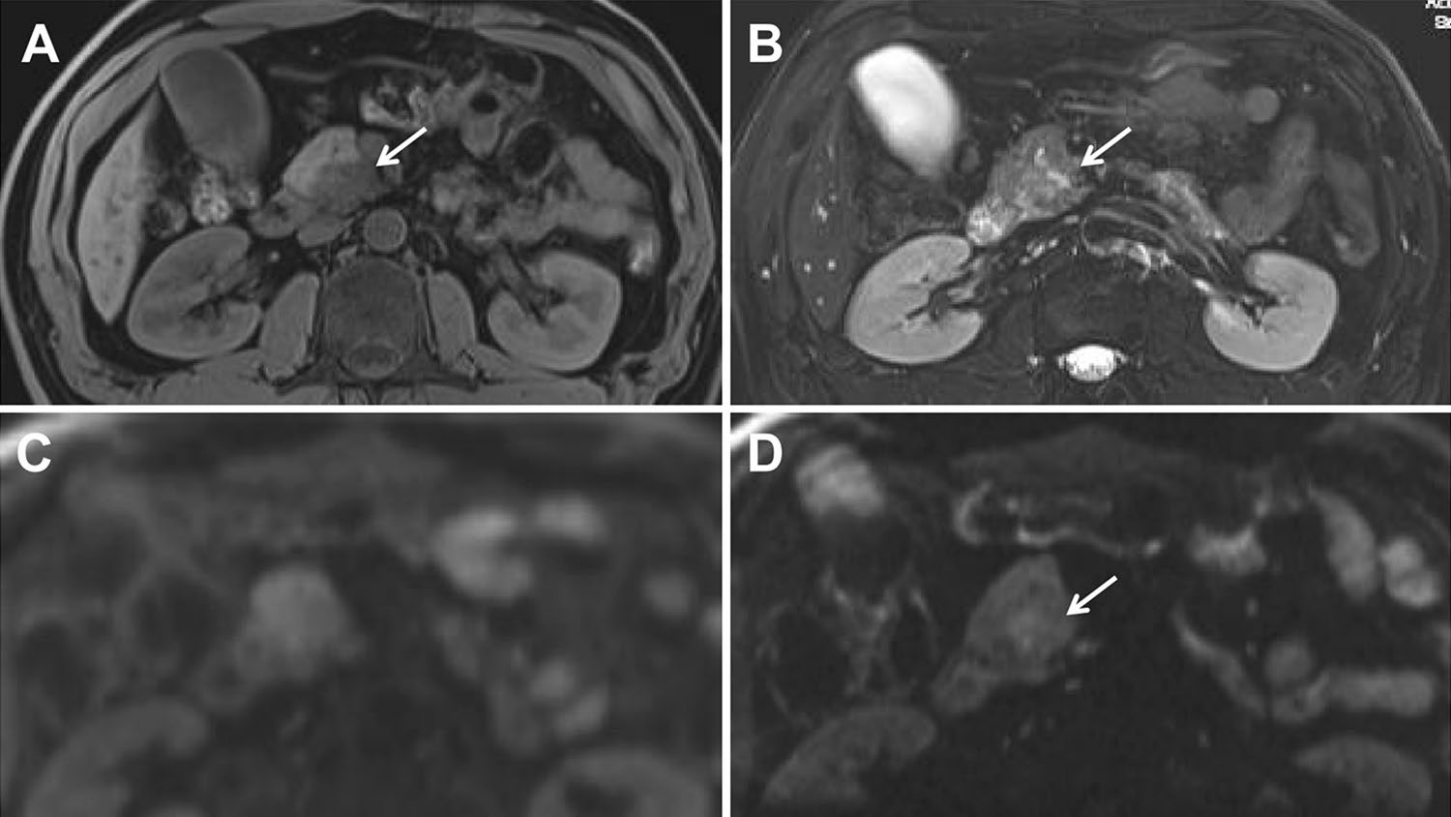
A 42-year-old man with pancreatic head carcinoma with a diameter of 19.0mm. **A** T1-weighted MR image and **B** T2-weighted MR image show a mass (arrow) in the pancreatic head. **C** c-EPI DWI shows no abnormality in the periampullary region **D** z-EPI DWI shows increased signal intensity of the lesion with better delineation and clear margin (arrow)

**Fig. 3:**
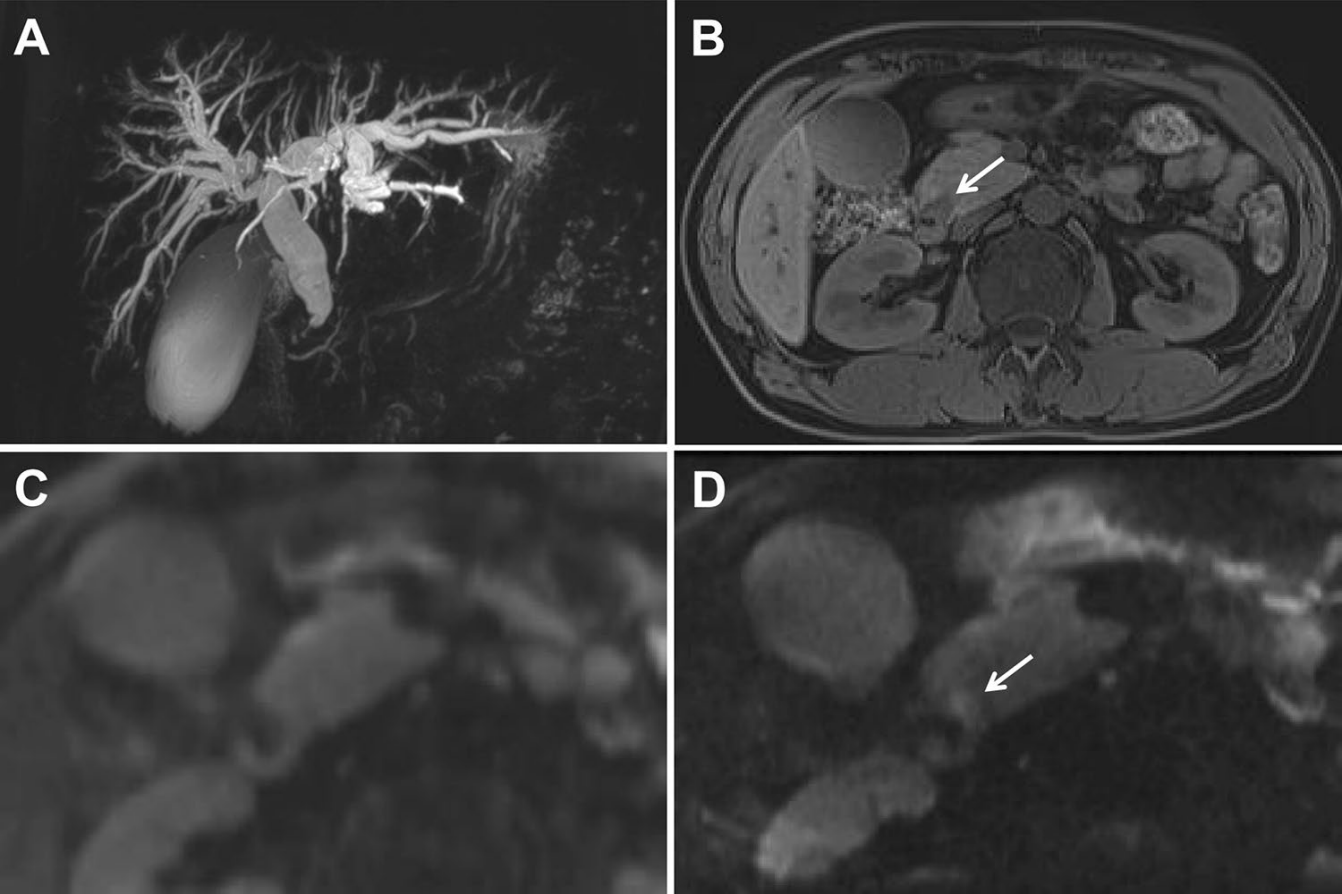
A 49-year-old man with distal common bile duct (CBD) carcinoma with a diameter of 9.9 mm. **A** MRCP shows marked bile duct dilatation with stenosis of the distal common bile duct (CBD) carcinoma. Main pancreatic duct dilatation was not noted. **B** T1-weighted MR image shows a hypointense nodule (arrow) in the periampullary region. **C** c-EPI DWI shows no abnormality in the periampullary region. **D** z-EPI DWI shows increased signal intensity of the lesion with better delineation

**Fig. 4:**
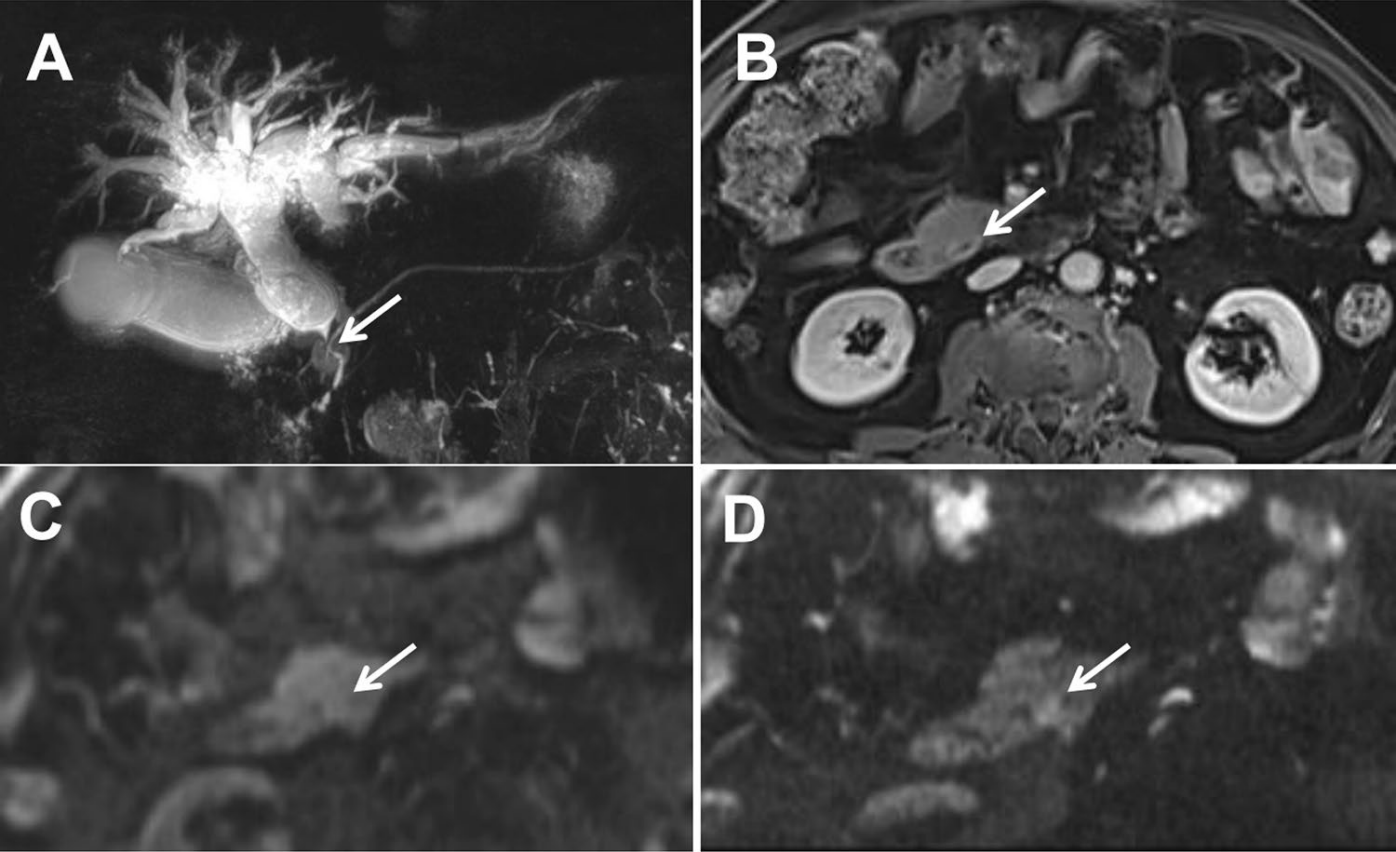
A 72-year-old man with distal common bile duct (CBD) carcinoma with a diameter of 15.3 mm. **A** MRCP shows marked bile duct dilatation with stenosis of the distal common bile duct (CBD) carcinoma. Main pancreatic duct dilatation was not noted. **B** T1-weighted MR delay phase image shows the thickened wall of the distal CBD with obvious enhancement. **C** c-EPI DWI shows no abnormality in the periampullary region. **D** z-EPI DWI shows increased signal intensity of the lesion with better delineation

**Fig. 5:**
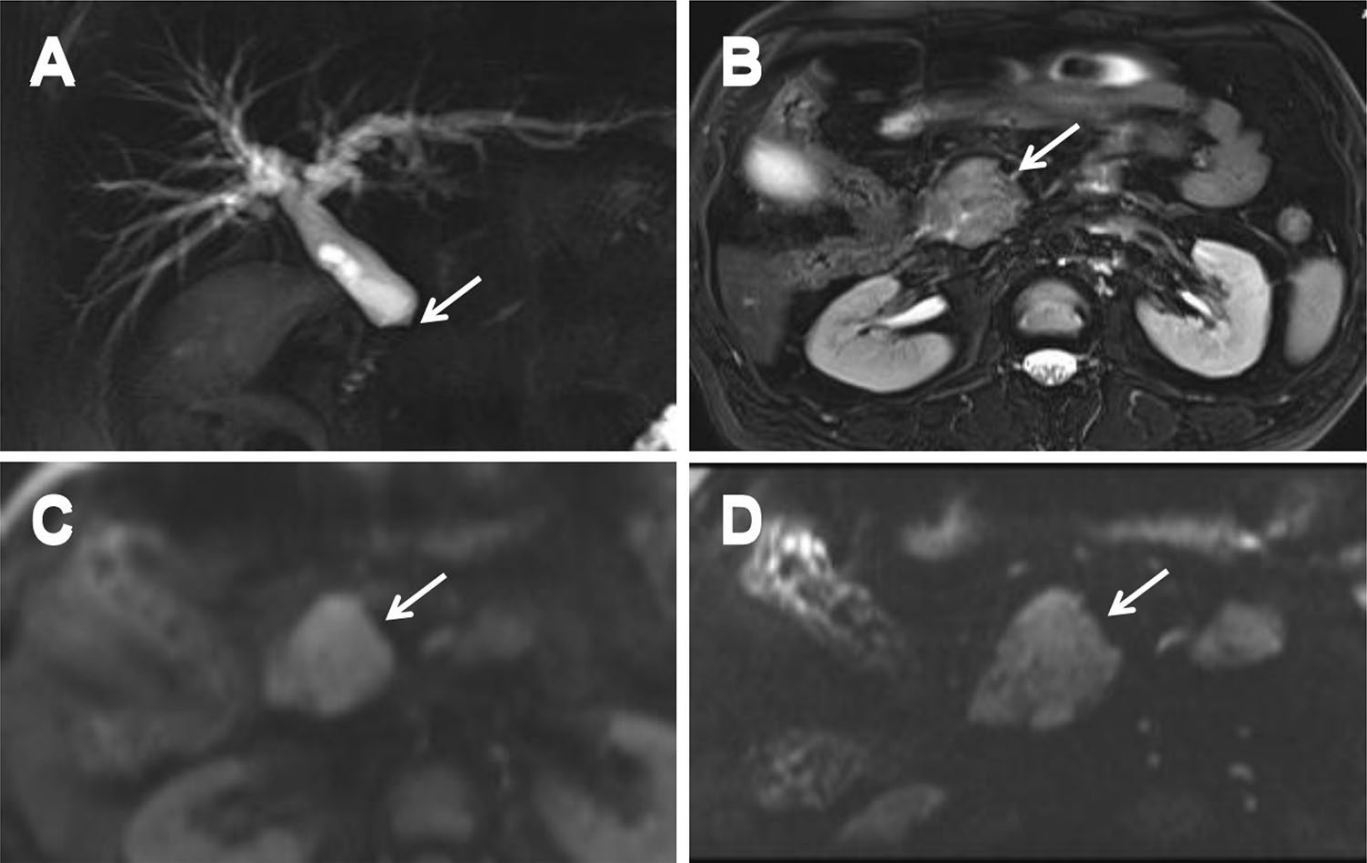
A 49-year-old man with autoimmune pancreatitis with a diameter of 37.3mm. **A** MRCP shows stenosis of the distal common bile duct (CBD). **B** T2-weighted MR image shows swelling of pancreas head. **C** c-EPI DWI shows no abnormality in the periampullary region. **D** z-EPI DWI also shows no abnormality in the periampullary region with better anatomic delineation

Most of periampullary malignancy on z-EPI DWI showed hyperintense signal for all lesions (33/36, 91.7%) and small lesions with diameters less than or equal to 20 mm (19/20, 95.0%). However, the hyperintense signal for all lesions and small lesions on c-EPI DWI was 69.4% (25/36) and 55.0% (11/20), respectively. The visual assessment of DWI in the all periampullary malignant lesions was increased from z-EPI DWI compared to c-EPI DWI (*P* = 0.023) (Figs 2, 3, 4). However, there were no statistically significant differences between the two image datasets for the small lesions (*P* = 0.45) (Table 5). For benign periampullary lesions, most on DWI showed isointense signal (Fig 5). The hyperintense signal on c-EPI DWI and z-EPI DWI was only 20.0% (3/15) for each.

**Table 5:** Visual assessment of c-EPI and z-EPI in the periampullary lesions (b=800 sec/mm^2^)

	Periampullary malignancy (*n* = 36)						Periampullary benignancy (*n* = 15)		
	All lesions *n* = 36			Small lesions (≤ 20 mm) *n* = 20					
	Isointense	Slightly hyperintense/significantly hyperintense	*Hyperintense (%)	Isointense	Slightly hyperintense/significantly hyperintense	*Hyperintense (%)	Isointense	Slightly hyperintense/significantly hyperintense	*Hyperintense (%)
z-EPI	3	12/21	91.7%	1	6/13	95.0%	12	2/1	20.0%
c-EPI	11	12/13	69.4%	9	3/8	55.0%	12	2/1	20.0%
*P* value			0.023			0.45			

*Rate of slightly hyperintense and significantly hyperintense lesions

*z-EPI* zoomed echo-planar imaging; *c-EPI* conventional echo-planar imaging

### Diagnostic accuracy scores analysis

Diagnostic accuracy scores were observed and compared between z-EPI DWI and c-EPI DWI, combined with MRCP. For all the malignant periampullary lesions and small lesions, the diagnostic accuracy scores were increased using the MRCP and z-EPI DWI combined set, compared to the MRCP and c-EPI DWI combined set (*P* < 0.05) (Table 6). However, there were no statistically significant differences between the two image data combined sets for benign periampullary lesions (*P* > 0.05).

**Table 6:** Comparison of diagnostic accuracy scores between the MRCP and c-EPI DWI combined set and the MRCP and z-EPI DWI combined set

Periampullary malignancy (*n* = 36)						Periampullary benignancy (*n* = 15)		
All lesions *n* = 36			Small lesions (≤ 20 mm) *n* = 20					
MRCP+ c-EPI DWI	MRCP+ z-EPI DWI	*P* value	MRCP+ c-EPI DWI	MRCP+ z-EPI DWI	*P* value	MRCP+ c-EPI DWI	MRCP+ z-EPI DWI	*P* value
4.00 ± 1.10	4.44±0.74	0.005	3.60±1.19	4.40±0.75	0.005	2.40±1.40	2.40±1.40	1.00

*z-EPI* zoomed echo-planar imaging; *c-EPI* conventional echo-planar imaging; *MRCP* magnetic resonance cholangiopancreatograph

**Fig. 6:**
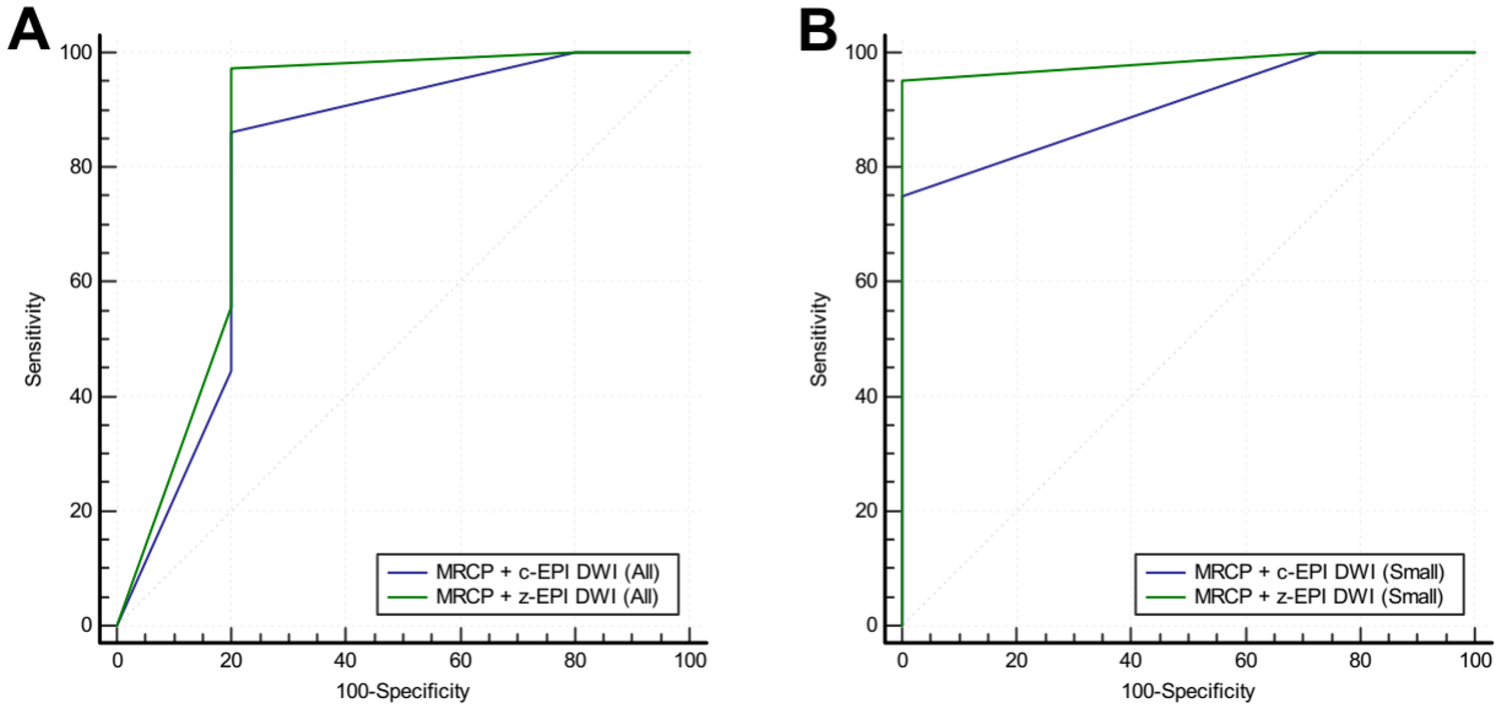
ROC curve for diagnostic accuracy in differentiating benign from malignant periampullary diseases between the MRCP and c-EPI DWI combined set and the MRCP and z-EPI DWI combined set. **A** For all the periampullary lesions , the area under the ROC curve (AUC) was significantly improved using the MRCP and z-EPI DWI combined set, compared to the MRCP and c-EPI DWI combined set (from 0.803 to 0.847) (*P* < 0.05). **B** For periampullary lesions with a diameter less than or equal to 20 mm, AUC was also improved using the MRCP and z-EPI DWI combined set, compared to the MRCP and c-EPI DWI combined set (from 0.909 to 0.982) (*P* < 0.05)

Diagnostic accuracy for detection and differentiation of periampullary malignant lesions from benign lesions significantly improved for the MRCP and z-EPI DWI combined set compared with MRCP and c-EPI DWI combined set (Fig 6). For all the periampullary lesions, the area under the ROC curve (AUC) was significantly improved using the MRCP and z-EPI DWI combined set, compared to the MRCP and c-EPI DWI combined set (from 0.803 to 0.847) (*P* = 0.0098). The diagnostic sensitivity of the MRCP and z-EPI DWI combined set was increased to 97.22% compared with MRCP and c-EPI DWI combined set (86.11%). However, the specificity of the MRCP and z-EPI DWI combined set and MRCP and c-EPI DWI combined set was the same (80.00%). For periampullary lesions with a diameter less than or equal to 20 mm, AUC was also improved using the MRCP and z-EPI DWI combined set, compared to the MRCP and c-EPI DWI combined set (from 0.909 to 0.982) (*P* = 0.0446). The diagnostic sensitivity of the MRCP and z-EPI DWI combined set was increased to 95.00% compared with MRCP and c-EPI DWI combined set (75.00%). And the specificity of the MRCP and z-EPI DWI combined set and MRCP and c-EPI DWI combined set was the same (100.00%).

### Quantitative ADC Measurements

There were no significant differences between c-EPI DWI and z-EPI DWI in the ADC values of periampullary malignant ((1.11±0.19)×10^-3^ mm^2^/s vs. (1.10±0.18)×10^-3^ mm^2^/s. *P* = 0.073) (n = 29) and benign lesions ((1.09±0.10)×10^-3^ mm^2^/s vs. (0.98±0.14)×10^-3^ mm^2^/s. *P* = 0.068) (n = 4), respectively.

## Discussion

Abdominal imaging is often disturbed by adjacent air in the gastrointestinal tract and the movement of abdominal organs and the aorta during scanning. Considering that z-EPI DWI uses two-dimensional spatial-selective radiofrequency pulses to obtain reduced volumes, z-EPI DWI potentially has advantages over c-EPI DWI. This technology can achieve higher resolution and reduced distortions without introducing unfolding artifacts [11, 21]. z-EPI DWI has previously been applied to certain organs including the pancreas, kidney, prostate, uterine, spinal cord, rectum, and head and neck regions [11–20]. In upper abdominal organs, reports of decreased FOV DWI mainly focus on the study of pancreas [11, 15]. However, most studies mainly evaluated the feasibility and superiority of images from the technical perspective while there are few reports focused on the clinical practice and disease diagnosis. To our knowledge, this is the first MRI study to evaluate the use and clinical application value of z-EPI DWI to study the periampullary region, including small-sized lesions.

Our results indicated that z-EPI DWI shows better delineation of the periampullary region with anatomic structure visualization and overall image quality, compared to c-EPI DWI. In addition, z-EPI DWI image artifacts were decreased, and the ADC values of periampullary malignant and benign lesions on z-EPI DWI were generally equivalent to those on c-EPI DWI. Our results were consistent with previous studies [11–13, 20]. Furthermore, we detected the ability of z-EPI DWI in delineating of the lesion conspicuity and lesion margin. Our study results showed that z-EPI DWI earned significantly higher scores for lesion conspicuity and lesion margins than c-EPI DWI, which will be helpful for accurately identifying lesion boundaries.

Thanks to the current advancement of MR technology, DWI of the abdomen has been more widely used to characterize benign and malignant tumors, particularly for detecting isointense or iso-attenuating periampullary malignant lesions. A small but important subset, namely, improved lesion conspicuity on DWI, is clinically significant. It was reported that 91% of ampullary carcinomas showed hyperintensity on DWI, whereas all benign cases showed iso-intensity, indicating that the addition of DWI to conventional MR imaging improves the detection of ampullary carcinoma when compared with conventional MR imaging alone [6]. In our study, 30.6% of periampullary malignancy showed iso-intensity on c-EPI DWI, which may be due to high proportion of small lesions. Conventional DWI may miss small lesions. The advantages of z-EPI DWI for lesion detection would likely be more substantial for smaller lesions. Our study further indicated that for lesions ≤ 20 mm, z-EPI DWI has better detection and delineation with lesion conspicuity, lesion margins, and diagnostic confidence compared with c-EPI DWI. Notably, the rate of hyperintense signal of all periampullary malignant lesions on z-EPI DWI was increased compared with that on c-EPI DWI, which may increase the diagnosis confidence when malignant lesions are not clearly visible on c-EPI DWI.

MRCP was widely used for the diagnosis of biliary obstructive lesions and is helpful in differentiating benign and malignant obstructions. However, MRCP alone has limited value. It was reported that combined DWI with MRCP can improve the diagnostic accuracy for differentiating malignant from benign strictures in the periampullary region. Diagnostic accuracy for malignant periampullary lesions improved after adding DWI [7]. Therefore, for the combined value of MRCP and z-EPI DW for periampullary lesions, we found that diagnostic accuracy was increased using the MRCP and z-EPI DWI combined set, compared to the MRCP and c-EPI DWI combined set. It was also suitable for small lesions. We confirmed the usefulness of z-EPI DWI as an added utility to MRCP for the diagnosis of periampullary lesions.

There are some limitations to this study. First, its retrospective nature may have presented a potential source of selection bias. Second, the small numbers of benign lesions were included in this study. Third, a quantitative analysis of the ADC values of few lesions were not performed as it was difficult to accurately measure because of their relatively small size. Fourth, the technology itself has the limitation of not capturing pathologies outside the zoomed FOV. c-EPI DWI was kept as a conventional clinical setting, while the z-EPI DWI was optimized for achieving better image quality. Therefore z-EPI DWI should be additionally performed on the basis of c-EPI DWI application.

## Conclusions

In conclusion, compared to c-EPI DWI, z-EPI DWI of periampullary carcinomas has an advantage in that it could lead to higher overall image quality as well as better anatomic structure visualization, lesion conspicuity, diagnostic confidence, and diagnostic accuracy. It would help radiologists better evaluate periampullary lesions in greater detail, particularly when they are small.

## Data Availability

The datasets used and/or analyzed during the current study are available from the corresponding author on reasonable request.

## Abbreviations

MRI: Magnetic resonance imaging
MRCP: Magnetic resonance cholangiopancreatography
z-EPI: Zoomed echo-planar imaging
c-EPI: Conventional echo-planar imaging
DWI: Diffusion-weighted imaging
T1WI: T1-weighted imaging
T2WI: T2-weighted imaging
